# The intestinal microbiome of inflammatory bowel disease across the pediatric age range

**DOI:** 10.1080/19490976.2024.2317932

**Published:** 2024-02-25

**Authors:** Máire A. Conrad, Kyle Bittinger, Yue Ren, Kelly Kachelries, Jennifer Vales, Hongzhe Li, Gary D. Wu, Frederic D. Bushman, Marcella Devoto, Robert N Baldassano, Judith R. Kelsen

**Affiliations:** a Division of Gastroenterology, Hepatology, and Nutrition, The Children’s Hospital of Philadelphia, Philadelphia, PA, USA; b Department of Pediatrics, Perelman School of Medicine, University of Pennsylvania, Philadelphia, PA, USA; c Department of Biostatistics and Epidemiology, Perelman School of Medicine, University of Pennsylvania, Philadelphia, PA, USA; d Division of Gastroenterology, Perelman School of Medicine, University of Pennsylvania, Philadelphia, PA, USA; e Department of Microbiology, Perelman School of Medicine, University of Pennsylvania, Philadelphia, PA, USA; f Institute for Research in Genetics and Biomedicine, Consiglio Nazionale delle Ricerche, Monserrato, CA, Italy; g Department of Translational and Precision Medicine, Università Sapienza, Rome, Italy

**Keywords:** Inflammatory bowel disease, very early onset inflammatory bowel disease, gut microbiome, intestinal microbiome, pediatrics, dysbiosis, microbial maturity

## Abstract

Dysbiosis is associated with pediatric and adult-onset inflammatory bowel disease (IBD), but the role of dysbiosis and the microbiome in very early onset IBD (VEO-IBD) has not yet been described. Here, we aimed to demonstrate the impact of age and inflammation on microbial community structure using shotgun metagenomic sequencing in children with VEO-IBD, pediatric-onset IBD, and age-matched pediatric healthy controls (HC) observed longitudinally over the course of 8 weeks. We found disease-related differences in alpha and beta diversity between HC and children with IBD or VEO-IBD. Using a healthy microbial maturity index modeled from HC across the age range to characterize their gut microbiota, we found that children with pediatric-onset IBD and VEO-IBD had lower maturity than their age-matched HC groups, suggesting a disease effect on the microbial community. In addition, patients with pediatric IBD had significantly lower maturity than those with VEO-IBD, who had more heterogeneity at the youngest ages, highlighting differences in these two cohorts that were not captured in standard comparisons of alpha and beta diversity. These results demonstrate that young age and inflammation independently impact microbial community structure. However, the effect is not additive in the youngest patients, likely because of the heterogeneous and dynamic stool microbiome in this population.

## Introduction

The pathogenesis of inflammatory bowel disease (IBD), including Crohn disease, ulcerative colitis, and IBD-unclassified, is complex and includes a dysregulated immune response to environmental factors in a genetically susceptible host. Studies have shown the diverse role that the intestinal microbiome plays in both pediatric and adult patients with IBD, ranging from specific pathogens, such as adherent invasive *E. coli*, to pathogenic communities, to a dysregulated immune response to commensal organisms. All of these factors alter the microbial community structure, also known as dysbiosis.^1–7^ In addition to increased pathobionts or pathogens, dysbiosis may include the loss of beneficial organisms and a decrease in the overall alpha diversity of the microbial community structure. Prior studies of dysbiosis associated with pediatric inflammatory bowel disease have demonstrated increased abundance in *Enterobacteriaceae, Pastueruellaceae, Veillonellaceae, Fusobacteriaceae* and decrease in *Bacteroidales, Clostridiales, Erysipelotrichaceae* and *Bifidobacteriaceae*.^1^ Furthermore, disease classification and location, phenotype, and severity have been associated with differences in the taxa and their abundances.^1,8,9^ Pro-inflammatory taxa that correlate with markers of disease activity include *Prevotella*, *Ruminococcus gnavus*, and Proteobacteria.^8–12^

One of the biggest challenges in understanding the microbial role in IBD, whether dysbiosis is a cause or effect of intestinal inflammation, remains unclear. This role and the time of onset of dysbiosis may be particularly relevant in the youngest children with IBD, as the microbial communities are inherently less diverse, immature, and change rapidly in the first few years of life, in parallel with the developing immune system.^13^ Previous studies have identified birth history, infant feeding, malnutrition, and antibiotics to be highly influential on the microbiome of healthy children; however, less is known about the role of inflammation in the microbiome during the first years of life.^14,15^

Very early onset IBD (VEO-IBD), IBD that presents from birth through 6 years of age, can have a distinct and more severe disease course than older patients with IBD. The identification of causal monogenic defects in some of these children over the last several years, has pointed to a stronger genetic contribution to the disease compared to older populations.^16^ Many of the identified defects are in genes associated with immunodeficiencies and genes involved in the integrity of the intestinal epithelial barrier.^17^ Despite the clear genetic role in VEO-IBD, the incidence and prevalence of VEO-IBD is rapidly increasing, suggesting an environmental and microbial impact on the disease.^18,19^ However, the influence of each of these disease drivers and how their interactions shape the development of VEO-IBD remains unclear. Studying these relationships, the evolving, dynamic microbiome, the immature, dysregulated immune response, and impaired barrier function at the mucosal interface in children with VEO-IBD may provide insight into some of these questions, and ultimately inform novel therapeutic approaches.

To characterize the microbial influences in VEO-IBD, we sought to differentiate the impact of age with its associated microbial stability and the impact of inflammation on the gut microbiome in children with very early onset (<7 years old) and pediatric IBD (7–18 years old). Based on prior studies, we hypothesized that the presence of inflammation is associated with dysbiosis in children with IBD, regardless of age. We also hypothesized that age will independently influence the microbial structure; therefore, the dynamic and developing microbiome will respond differently to inflammation in children with VEO-IBD than in those with pediatric IBD.

To accomplish these aims, we studied the impact of inflammation on the fecal microbiome in patients with VEO-IBD and pediatric IBD and compared them to the fecal microbiome of healthy controls over 8 weeks using shotgun metagenomic sequencing. In addition, to characterize the effects of age on microbial communities, we assessed the microbial maturity in all four cohorts’ samples.

## Results

### Study population and disease assessment

**Figure 1.**
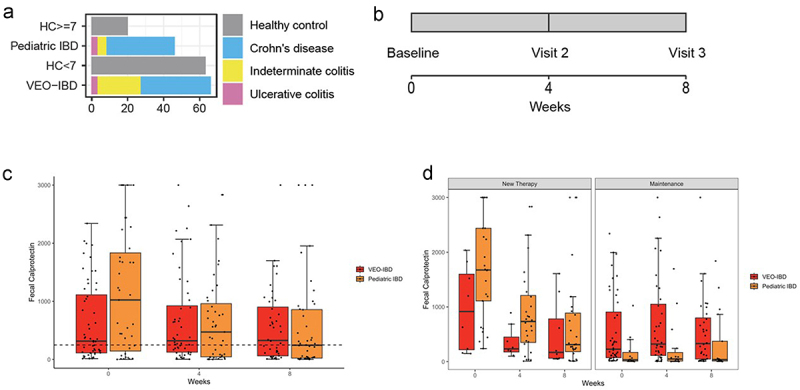
(a) Numbers of study participants by study group and by diagnosis. (b) Sample collection timeline. (c) Fecal calprotectin measurements at 3 study visits in stool samples of pediatric IBD (orange) and VEO-IBD (red) patients. The dotted line indicates a cut off of 250ug/g, below which was consistent with lower disease activity. Boxplot demonstrates median and interquartile values, and whiskers are minimum and maximum values. (d) Fecal calprotectin measurements over 3 study visits by disease cohort.. Left panel includes patients who started new therapy during the study and demonstrates decrease trend in calprotectin for 8 weeks, which was not seen among patients on maintenance therapy (right panel).

A total of 195 subjects were included in this analysis: 66 with VEO-IBD (all diagnosed <6 years old, at time of sampling 58 were <7 years old and 8 ≥ 7 years), 46 with pediatric IBD, and 83 age-matched healthy controls (HC) (63 were <7 years old and 20 ≥ 7 years old at the time of enrollment) (Figure 1(a)). Stool samples were collected at week 0 (baseline), week 4 (visit 1), and week 8 (visit 2) for disease assessment by calprotectin and microbiome profiling (Figure 1(b)). There were 8 VEO patients >7 years old at enrollment who provided only one sample and not included in the longitudinal analyses.

The demographic and clinical characteristics are detailed in Tables 1 and 2. Differences in disease characteristics were expected based on known differences in clinical phenotype between VEO-IBD and pediatric IBD. At the time of baseline sample collection, 35% of the VEO-IBD cohort and 59% of the pediatric IBD cohort were newly diagnosed and treatment-naïve. Therapies initiated after the baseline visit and continued through the 8 week follow-up period included anti-TNF-α antibody therapy (infliximab and adalimumab), aminosalicylates, and corticosteroids (Table 2). Forty-three (65%) children with VEO-IBD were on stable maintenance therapy during the study, including aminosalicylates, immunomodulators, anti-TNF-α antibody therapy, steroids, and exclusive enteral nutrition therapy. Nineteen children (41%) with pediatric IBD were on stable maintenance therapy during the study, including aminosalicylates, immunomodulators, and anti-TNF-α antibody therapy. Eight HCs less than 7 years old and 26 children with VEO-IBD were treated with antibiotics during the study window, and this exposure was recorded at the time of stool collection. No subjects in the pediatric IBD cohort or older HC cohort were exposed to antibiotics for at least 90 days prior to or during the study. Use of probiotics, iron supplements, and proton pump inhibitors are also recorded in Table 2.

**Table 1.** Clinical characteristics of all study groups.

	Healthy Controls		VEO-IBD		Pediatric IBD
	≥7 years old	<7 years old	Samples Collected <7yo	Samples Collected ≥7yo	
N, subjects	20	63	58	8	46
No. of samples collected	60	189	151	8	133
Male (%)	9 (45)	32 (51)	41 (71)	6 (75)	30 (65)
Age at Sample Collection, y, mean (SD)	12.44 (3.60)	3.58 (1.68)	4.31 (1.76)	10.54 (2.63)	14.28 (2.92)
Age at Diagnosis, y, mean (SD)	-	-	3.39 (1.50)	4.24 (1.80)	12.66 (2.66)
Ethnicity = Hispanic/Latinx	2 (10)	9 (14)	3 (5)	1(13)	4 (9)
Race White Black Asian >1 race Declined	14 (70) 2 (10) 1(5) 1(5) 2 (10)	43 (68) 11(17) 0 (0) 8 (13) 1 (2)	50 (86) 1 (2) 7 (12) 0 (0) 0 (0)	8 (100) 0 (0) 0 (0) 0 (0) 0 (0)	35 (76) 8(17) 0 (0) 3 (7) 0 (0)

**Table 2.** Clinical characteristics of patients with inflammatory bowel disease.

	VEO-IBD	Pediatric IBD	P-value
n	66	46	
*Type of IBD, n (%)*			0.010
Crohn’s Disease	39 (59.1)	38 (82.6)	
IBD-unclassified	24 (36.4)	5 (10.9)	
Ulcerative Colitis	3 (4.5)	3 (6.5)	
***Paris Classification of Pediatric IBD***			
*Crohn disease: Lower GI Disease location, n (%)*			0.001
Ileal (L1)	3 (8)	9 (24)	
Colonic (L2)	24 (62)	9 (24)	
Ileocolonic (L3)	11 (28)	18 (47)	
None	1 (2.5)	2 (5)	
*Crohn disease: Upper GI Disease location, n (%)*			<0.001
L4a	8 (21)	17 (44)	
L4b	0	3 (8)	
L4a/L4b	3 (8)	4 (11)	
None	28 (72)	14 (37)	
*Crohn disease behavior, n (%)*			0.014
Inflammatory (B1)	37 (95)	31 (82)	
Stricturing (B2)	1 (1.5)	5 (13)	
Stricturing and Penetrating (B2/B3)	1 (3.0)	2 (5)	
*Ulcerative colitis/IBD-U extent, n (%)*			0.027
Left-sided colitis (E2)	2 (7)	3 (38)	
Extensive colitis (E3)	4 (15)	0 (0)	
Pancolitis (E4)	20 (74)	5 (62)	
None	1(4)	0 (0)	
***IBD Treatments***			
*Newly diagnosed, n (%)*	23 (34.8)	27 (58.7)	0.021
*Therapies newly initiated during the study, n (%)*			
Aminosalicylates	9 (13.6)	13 (28.3)	0.094
Steroids	5 (7.6)	16 (34.8)	0.004
Immunomodulators	5 (7.6)	2 (4.3)	0.766
Biologic	6 (9.1)	15 (32.6)	0.004
Exclusive enteral nutrition therapy	1 (1.5)	0 (0)	1
*Maintenance Therapies, n (%)*			
Aminosalicylates	31 (47)	4 (8.7)	<0.001
Steroids	12 (18.2)	0 (0)	0.006
Immunomodulators	14 (21.2)	6 (13.0)	0.390
Biologic	11 (16.7)	17 (37.0)	0.027
Exclusive enteral nutrition therapy	10 (15.2)	0 (0.0)	0.015
*Other Medications and Supplements, n (%)*			
Proton Pump Inhibitor	6 (9.1)	1 (2.2)	
Probiotics	6 (9.1)	4 (8.7)	
Iron supplements	4 (6.1)	2 (4.3)	
*Fecal calprotectin, ug/g (mean (SD))*			
Baseline visit	623.54(668.42)	1152.33 (1057.68)	0.004
Visit 2	688.45(783.42)	677.10 (769.88)	0.945
Visit 3	568.68 (631.33)	569.96 (825.91)	0.994

Fecal calprotectins were obtained at each time point for the subjects in the VEOIBD and pediatric IBD cohorts, as a measure of disease activity (Figures 1(c,d)). Calprotectin samples were obtained from the same stool samples that were sequenced. Fecal calprotectin levels were elevated (normal defined as <250 µg/g) in 46% of both VEO-IBD and pediatric IBD groups, indicating active disease (Table 2, Figure 1(c)). At baseline, calprotectin levels were higher in the 50 newly diagnosed patients initiating new therapy compared to the 62 patients on stable maintenance therapy (*p* = 10^−9^) (Figure 1(d)). Fecal calprotectin levels then declined among those starting new therapy (*p* = 0.0001) over the 8-week study window (Figure 1(c)). Among the 62 patients with IBD on maintenance therapy, calprotectin levels did not change significantly during the study window (*p* = 0.9). However, calprotectin levels remained higher in the VEO-IBD cohort as compared to the pediatric IBD cohort on maintenance therapy throughout the study (*p* = 0.03) Figures 1(c,d). Antibiotic use did not have a statistically significant effect on calprotectin levels (antibiotics were only used in the VEO-IBD subcohort) (*p* = 0.1).

In addition to analyzing fecal calprotectin, we quantified the human DNA percentage in the shotgun metagenomic sequencing data as a biomarker for cell turnover, neutrophil activity, and deposition of dead cells in the gut lumen. We previously found an association between human DNA and inflammation, with higher percentages of human DNA detected in the stool of pediatric IBD patients and in pediatric IBD patients with *C. difficile* infection.^3^ Here, we again found that higher human DNA percentage was associated with disease (Supplemental Figure 1). Human DNA percentage was increased in stool samples from all IBD patients compared to HC (*p* = 10^−9^) but was not different between VEO-IBD and pediatric IBD patients (*p* = 0.08). In patients with IBD, it decreased over time among those who started new therapies (*p* = 0.009). Therefore, human DNA was positively correlated with fecal calprotectin (Spearman’s ρ = 0.46, *p* = 10^−6^ at baseline), in accordance with our previous observations.

*Differences in gut microbiome composition among VEO-IBD, pediatric IBD, and healthy controls*

**Figure 2.**
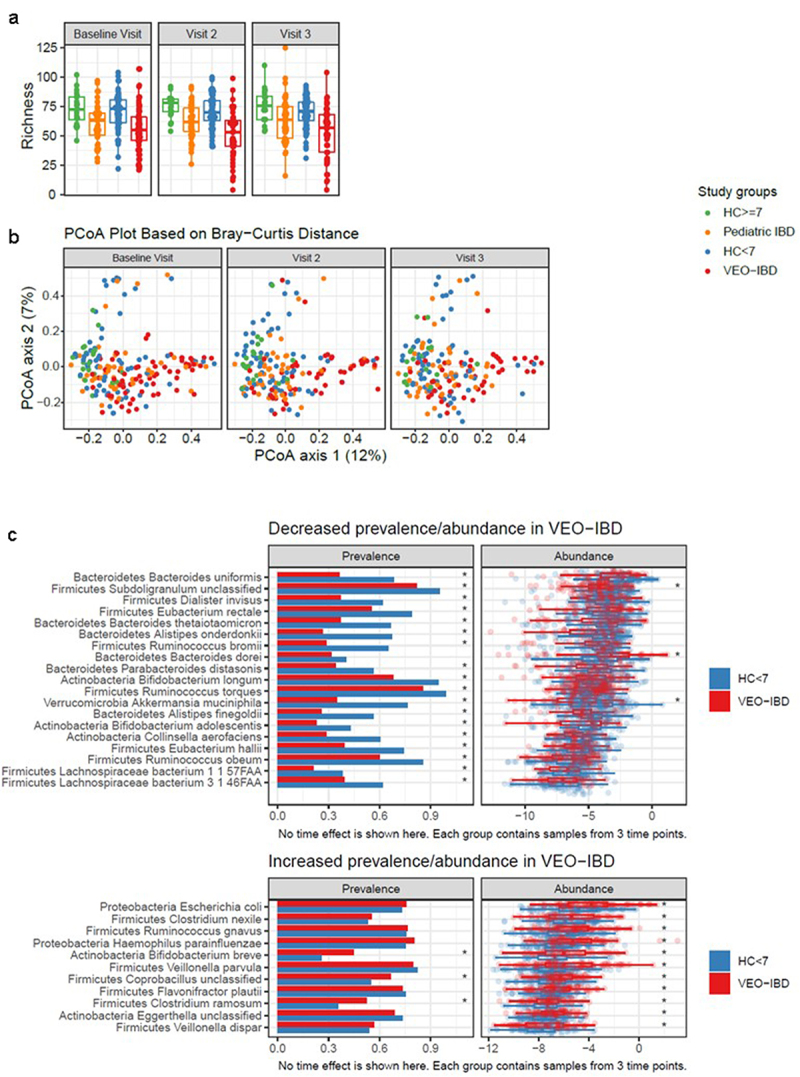
Comparison of fecal microbiota composition among study groups. samples are color coded to indicate the four study groups (green: HC ≥ 7, orange: pediatric IBD, blue: HC < 7, red: VEO-IBD). (a) Species richness at 3 study visits colored by study group. (b) Principal coordinate analysis of based on Bray-Curtis dissimilarity assessed using microbiome data. Samples collected at baseline visit, visit 2 (4 weeks), and visit 3 (8 weeks) are shown in separate facets. (c) Comparison of taxa prevalence and abundance in VEO-IBD and HC <7 years old. Top panel: taxa with significantly lower prevalence or abundance in VEO-IBD samples than HC < 7. Bottom panel: taxa with significantly increased prevalence and/or abundance in VEO-IBD compared to HC < 7. Asterisks demonstate comparisons with p < 0.05.

The gut microbiome of 195 subjects (540 total samples) was profiled by shotgun metagenomic sequencing. We first assessed microbial alpha diversity using species richness and found a decrease in VEO-IBD (*p* = 0.004) and pediatric IBD patients (*p* = 0.006) compared with corresponding HCs (Figure 2A). There was no difference in richness between the VEO-IBD and pediatric IBD groups (*p* = 0.1) (Figure 2A). Within disease subsets, there was also no difference among patients who started new therapy compared to those on stable maintenance therapy (*p* = 0.6) or throughout the study window (*p* = 0.5). Similarly, we found a significant decrease in Shannon Diversity (SD) in the stool of patients with VEO-IBD (*p* < 0.0001) and pediatric IBD (*p* = 0.0009) compared to their age matched HC cohorts (Supplemental Figure 2). However, as with richness, there was no significant difference in SD between the VEO-IBD and pediatric IBD groups (*p* = 0.21). Changes in richness and SD did not follow the patterns of calprotectin during this study; thus, despite improvement in calprotectin and disease activity in patients on new therapy over time, alpha diversity as measured by richness and SD were not restored.

As it is well established that antibiotic use affects the microbial community structure in the gut, we wondered whether differences in the type and degree of antibiotic exposure, or its interaction with disease, may have a particular impact on the alpha diversity of the gut microbiome in VEO-IBD. We found that, although antibiotic use did not significantly lower the richness among the HCs on antibiotics during the 8-week study window, it was associated with a substantially lower richness in VEO-IBD (*p* = 3×10^−6^). We obtained similar findings in the comparison of Shannon diversity between cohorts with antibiotic exposure.

The microbial community dissimilarity between samples was measured using the Bray-Curtis distance (Figure 2(b)). Following the approach of the Integrative Human Microbiome Project,^20^ we quantified the level of dysbiosis in each sample by computing dissimilarity to samples from healthy children aged ≥7 years; thus, the further the distance, the more dysbiotic the sample. Our analysis indicated that IBD was associated with increased dissimilarity from HCs (*p* = 5×10^−5^) after adjusting for age group and antibiotic exposure. In addition, dissimilarity was associated with younger age (*p* = 0.0001). Our linear mixed-effects model indicated that children in the VEO-IBD group did not have increased dissimilarity beyond the contributions of age and IBD diagnosis, that is, the interaction term between age and disease groups was not statistically significant (*p* = 0.6). The level of dissimilarity did not change over time for any of the cohorts (*p* = 0.4), and as seen with the measures of alpha diversity, in both the IBD cohorts it was not associated with new versus maintenance therapy (*p* = 0.6), in contrast to our findings with calprotectin. Rather, we found that the Bray Curtis distance to HCs remained steadily increased in pediatric IBD patients over the study period, even as inflammation decreased. To further demonstrate the differences in temporal stability, we compared within-subject Bray-Curtis distances at all 3 time points to assess stability over time (Supplemental Figure 3). VEO-IBD patients exhibited greater instability relative to HC <7 years (*p* = 0.003), but this was not seen between pediatric IBD patients and HC ≥7 years (*p* = 0.053), although the direction of the coefficient was consistent with younger patients.

**Figure 3.**
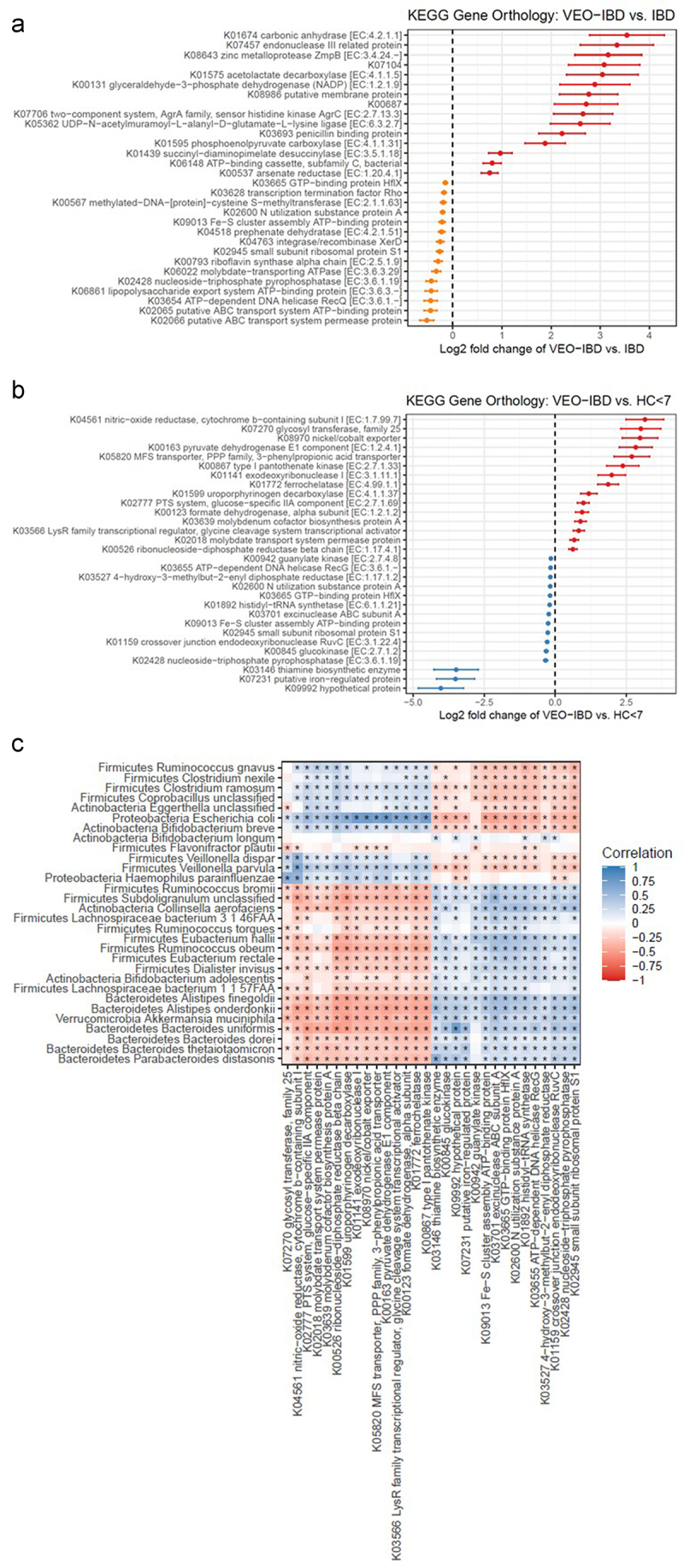
Ratio of log2 transformed KEGG ortholog abundances between VEO-IBD and pediatric IBD (figure 3a) and VEO-IBD and healthy controls <7 years old (figure 3B). positive values indicate higher abundance in VEO-IBD (in red), and negative values indicate higher abundance in pediatric IBD (in orange) and in HC < 7 (in blue). (c) Correlation of top 30 most abundant gene orthologs with top 30 significantly different taxa detected in VEO-IBD. heatmap indicates degree of correlation with blue positive and red negative and asterisk denotes significance. the asterisks within the heat map indicate a *p* value of < 0.05.

Antibiotic exposure increased the dissimilarity of the VEO-IBD group from HCs without antibiotics as expected (*p* = 0.001) but did not increase dissimilarity between HCs with antibiotic exposure and HCs without antibiotic exposure (*p* = 0.5). This could be related to host factors, the difference in duration of antibiotic treatment, or choice of antibiotics.

Next, we characterized taxonomic differences in the microbiome of VEO-IBD patients relative to HC aged <7 years. A total of 81 species were compared between study groups after excluding species that were only present in <20% of the samples (Supplemental Table 1(a)). We identified 19 species that showed decreased prevalence or abundance in the VEO-IBD group and 11 species that showed increased prevalence or abundance (Figure 2(c)). Gut-associated *Clostridia* and *Bacteroidetes* species comprised the majority of those that were decreased in VEO-IBD. Two species of *Bifidobacterium*, *B. longum* and *B. adolescentis*, as well as *Akkermansia muciniphilia*, were also decreased. Of the 11 species that were increased in VEO-IBD, most have been previously associated with gut dysbiosis or with IBD, such as *Ruminococcus gnavus, Escherichia coli*, and *Veillonella parvula*.^1,21,22^ We also demonstrated these findings in our comparison of pediatric IBD to age matched HC (Supplemental Figure S4, Supplemental Table S1b). Interestingly, both disease cohorts had a greater abundance of *E. Coli* than their healthy counterparts, in addition to several similar taxa with decreased prevalence and/or abundance, including commensal organisms such as *Eubacterium rectale, Alistipes onderdonkii, and Alistipes finegoldii* from healthy controls.

Because our taxonomic analysis included subjects in both healthy and disease cohorts with current antibiotic exposures at sample collection, our models included an additional term to estimate relative abundance differences associated with antibiotic use. Among the 11 species that increased in VEO-IBD, we found no association with antibiotics in *E. coli*, *C. nexile, B. breve*, unclassified *Coprobacillus*, or *C. ramosum*, but we found an association between antibiotics with *R. gnavus* (q = 0.001), *H. parainfluenzae* (q = 0.007), *V. parvula* (q = 0.002), *F. plautii* (q = 0.001), unclassified *Eggerthella* (q = 0.043), and *V. dispar* (q = 0.047).

We then compared children with VEO-IBD to those with pediatric IBD (supplemental Table 1(c)) and identified four species that were more abundant in the VEO-IBD group than in the pediatric IBD group: *Clostridium nexile* (q = 0.001), *Haemophilus parainfluenzae* (q = 0.014), *Clostridium ramosum* (q = 0.03), and unclassified *Veillonella* (q = 0.03) none of which are known to be pathogenic in the gut. Because no pediatric IBD patients on antibiotics were enrolled in this study, we excluded VEO-IBD patients who were on antibiotics from the comparison with the pediatric IBD group.

### A functional profile of the gut microbiome in VEO-IBD

Next, we sought to identify potential differences in the functional composition of the fecal microbiome in children. The relative abundances of bacterial gene orthologs were compared between children with VEO-IBD and pediatric IBD and between the VEO-IBD and HC < 7 groups. (Supplemental Tables 2A and 2B) We identified 181 genes that differed between VEO-IBD and pediatric IBD patients (q < 0.05). Among the genes that were most increased in VEO-IBD, several coded for metal-binding proteins or surface-associated proteins, indicating a possible role in virulence or response to host inflammation. (Figure 3(a)). Our gene function analysis identified 679 orthologs that differed between VEO-IBD and HC < 7yo, where again we observed that genes associated with transition metal transport and binding were among those that increased the most in VEO-IBD (Figure 3(b)). Similar comparison between HC > 7yo and pediatric IBD of the 30 bacterial gene orthologs with the highest abundance is shown in Supplemental Figure S5 (and entire list at Supplemental Table S2(c)).

Upon integrating the functional and microbial analyses, we found that the 11 taxa increased in VEO-IBD (listed above) were positively correlated with the gene abundance pathways most frequently detected in VEO-IBD samples (Figure 3(c)). The strongest positive correlations were observed between *E. coli* and *E. coli*-specific orthologs, including formate dehydrogenase, type 1 pantothenate kinase, MFS transporter, and LysR family transcriptional regulator.

### A microbiota maturity index for children with IBD

**Figure 4.**
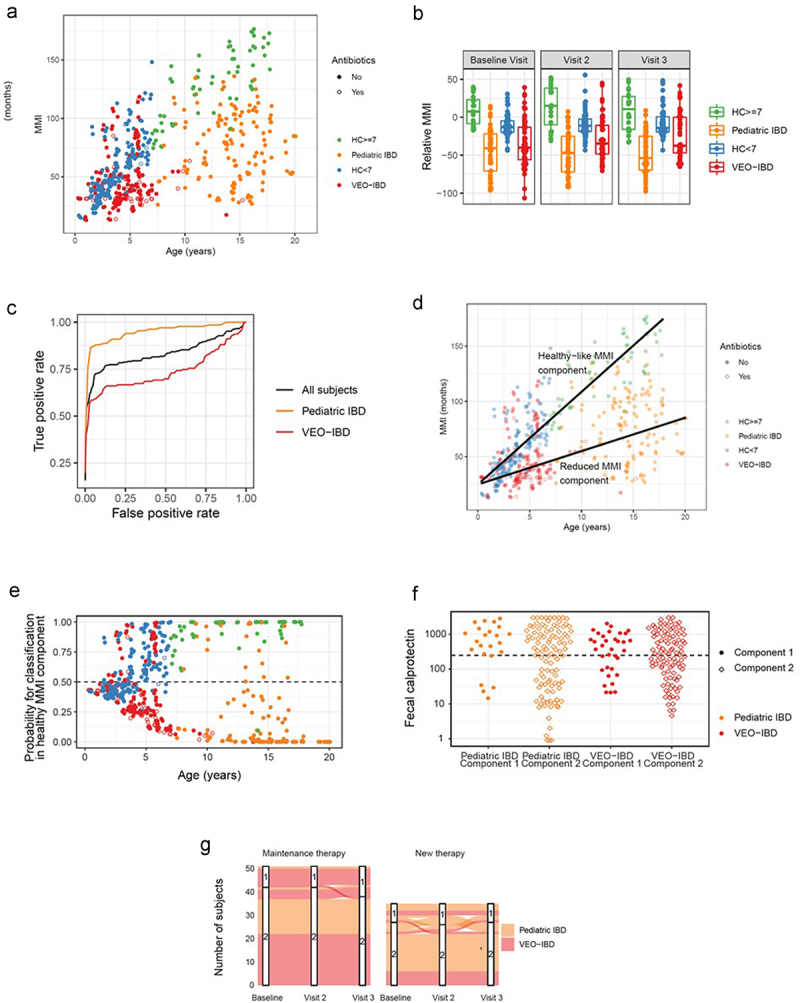
The microbiota maturity index (MMI) was developed from healthy controls in order to identify the taxa most associated with health at different ages. (a) Each sample’s MMI was plotted against chronological age and colored by study group. samples with recent/current antibiotic exposure are denoted with open circle. With increased chronological age, there is a divergence in MMI between older healthy controls and pediatric IBD, not seen in younger children. (b) The relative MMI is lowest among patients with pediatric IBD and more heterogeneous among children with VEO-IBD than healthy controls. (c) Receiver operating characteristic (ROC) curve analysis demonstrates the efficacy of relative microbial maturity index to predict disease status. RMMI is a good predictor of disease status overall with AUC = 0.83. It performed better at predicting pediatric IBD status (AUC = 0.93) than VEO-IBD (AUC = 0.74). (d) Figure 4A plot of MMI with the addition of lines fit to two component model, including healthy-like MMI component fit among the HC and reduced-MMI component. (e) Each sample’s probability of being in healthy-like MMI component 1 vs reduced MMI component 2. There is greater separation with increasing age suggesting the model can discern samples more effectively in the older age groups. (f) Fecal calprotectin grouped by disease group and component demonstrated that despite differences in microbial communities in each component there were not differences in disease activity as measured by calprotectin. (g) There were limited numbers of patients whose samples switched component groups between healthy and non-healthy components during follow up. Those subjects that did see more variability were more likely to have been started on new therapy during the study window than those on stable maintenance therapy.

To further delineate the differences between VEO-IBD and pediatric IBD with age-appropriate HCs along the age spectrum, we used our data to compute a microbiota maturity index (MMI) following the approach of Subramanian et al.^23^ (Figure 4(a)). The taxa identified for this model are listed in supplemental Table S3. When applying the model to IBD, we included stool samples from eight patients with VEO-IBD obtained later in their disease course (sampled at age 8–17 years old) to further characterize the effects of age and disease duration on the gut microbiome. The absolute MMI ranged from 12.9 to 136 months among all IBD patients. When comparing the two HC cohorts with the IBD cohorts, we observed that the slope of microbiome maturity versus age was lower for IBD samples than for HCs (*p* = 10^−16^).

To better understand the deviation from healthy MMI in children with IBD, we fit the MMI to the healthy control samples as a function of age and analyzed each sample relative to the age corrected MMI of healthy controls, hereafter referred to as Relative MMI (RMMI) (Figure 4(b)). The mean RMMI for the pediatric IBD group was reduced by 55 months relative to that of the HC (*p* = 10^−10^). In the VEO-IBD cohort, the mean RMMI was reduced by 25 months (*p* = 10^−6^). RMMI did not systematically increase or decrease between visits, suggesting that RMMI differences represent a long-term, rather than short-term, departure from the pattern of gut microbiome maturation in healthy children. Using ROC curve, we also demonstrated that RMMI was a good predictor of disease status with area under the curve (AUC) = 0.83, but it was an excellent predictor of disease status in the older children with AUC = 0.95, while AUC was 0.74 in VEO-IBD cohort (Figure 4(c)). Species-level correlations of MMI-specific taxa using a SparCC network map demonstrated that those taxa used in the MMI that are highly correlated in healthy controls with age were not the same correlations in IBD/VEO-IBD patients (Supplemental Figure S6). These correlations were disrupted in the diseased cohort; and therefore, the difference in the RMMI is not just from an expected decrease in correlated taxa but instead different correlations all together.

Having observed that the departure from RMMI was more extreme in older children with IBD, we hypothesized that gut microbiome maturity may lie along a long-term trajectory that is delayed relative to that of healthy children. To examine this hypothesis quantitatively, we developed a two-component model using the MMI depicted in Figure 4(a), where the first component of the model was fit among the HCs, and the slope of the second component was determined using an expectation-maximization algorithm (Figure 4(d)). Once we established the two components, we computed the posterior probability for each sample’s membership in component 1 (healthy-like MMI component) or component 2 (reduced-MMI component) (Figure 4(e)). For children under 5 years old, near the point of convergence for the two components, the posterior probability did not indicate a strong preference for membership in either component (85% of samples with posterior probability between 0.25 and 0.75). However, for children over 5 years old, the model indicated a strong preference for membership in one of the components (83% of samples with posterior probability above 0.75 for one component).

When comparing the samples from the two components, the characteristics of IBD and VEO-IBD samples that fell into the reduced-MMI component included lower richness (pediatric IBD, *p* = 0.02; VEO-IBD, *p* = 8×10^−5^) and Shannon diversity (pediatric IBD, *p* = 6×10^−5^ VEO-IBD, *p* = 9×10^−5^). Some pediatric IBD patients in the reduced-MMI component tended to have a longer disease duration than those pediatric IBD subjects in the healthy-like MMI component (*p* = 0.08). When comparing calprotectin levels between the healthy-like MMI and reduced-MMI components, there was no difference in either VEO-IBD (*p* = 0.6) or pediatric IBD (*p* = 0.4) (Figure 4(f)). Finally, we analyzed how often samples from the same subject switched between MMI components between visits (Figure 4(g)). Overall, the predicted components were stable within subjects. When switching occurred, we observed more among the pediatric IBD and VEO-IBD patients on new therapy than on maintenance therapy. Thus, our analysis of microbiome maturity in VEO-IBD and pediatric IBD patients underscores a framework of persistent microbiome differences spanning a wide range of ages, despite short-term changes in inflammation that are associated with disease treatment.

## Discussion

This is one of the first descriptions of the intestinal microbiome of children with IBD from early childhood through adolescence, including VEO-IBD and pediatric IBD.^1–4^ Studies of the intestinal microbiome in pediatric patients can be challenging due to the dynamic nature of the microbial community structure early in life and to a lesser degree throughout most childhood. It has been well established that in the first 3 years of life, the evolving gut microbiome is unstable, constantly influenced by environmental factors such as birth mode, geography, weaning breastfeeding, dietary changes and antibiotics.^24–26^ Beyond the first 3 years of life, little is understood about the developing gut microbiome; however, several studies have shown that the effects of maturation can persist throughout childhood. For example, the microbial structure in older children and adults can differ in the distribution and relative abundance of certain genera, including decreased aerobes and facultative anaerobes, but with increased obligate anaerobes in adults.^13,24,27^ There is also some evidence for greater interpersonal variation in bacterial composition and greater temporal variation in younger children than in adults, which may affect health later in life.^24,26,28^ Here, we studied differences in the gut microbiota in younger and older children, with and without IBD, in order to differentiate the effects of age and inflammation using dissimilarity in microbial communities as a marker of dysbiosis.

We identified increased dissimilarity among the combined younger cohorts (VEO-IBD + HC ≤ 7) as compared with the combined older cohorts (IBD + HC > 7). This may highlight the impact of instability of the microbiome in young age on a dysbiotic state. We also saw that inflammation and disease status affected dysbiosis, demonstrated by the significant difference in dissimilarity between IBD patients and age-matched HC cohorts. However, while age and inflammation independently impact the microbial community structure, consistent with prior reports,^1,3,24,27^ there was no additive effect in the youngest patients with disease in this cohort. This may be due to the inherent and constant variation and different “commensal” community in the developing microbiome in young children, that is distinct from the variation caused by inflammation. It is also likely that our current methodology cannot distinguish between the instability and inflammation.

To further characterize the effect of inflammation on the fecal microbiome in both pediatric IBD and VEO-IBD patients, we quantified the specific taxa that accounted for these differences, including *C. nexile, H. parainfluenzae, C. ramosum*, and unclassified *Veillonella*. While a multitude of studies have attempted to understand whether specific microbes or communities of microbes are present in patients with IBD versus healthy individuals, much remains unclear. For example, dysbiosis in patients with IBD has not been consistently detected or found to be clinically relevant or predictive of prognosis.^1,3,29^ Similar findings were observed in this study, as calprotectin, a surrogate marker of intestinal inflammation and disease activity, improved while the markers of dysbiosis, including BC distance, richness, and SD, did not significantly change over 8 weeks. This was true even in the patients with active disease at baseline who initiated new therapies and had the most significant change in calprotectin levels over the study window. Together, these findings support the notion that dysbiosis is not directly associated with disease activity, as measured by clinical indices and calprotectin.

The inherently dynamic nature of IBD itself, like the microbiome in young children, may contribute to the difficulty in characterizing specific trends. A longitudinal study of the gut microbiome in an adult IBD population demonstrated persistent fluctuations in the microbiome of patients with IBD. Similarities were detected between IBD subjects and healthy controls; however, this similarity was only temporary. In some instances, this form of instability was potentially indicative of a pathologic process.^5^ Pediatric studies have also demonstrated interindividual variation in alpha and beta diversity among patients with IBD, but these studies have not accounted for differences in age.^30^ However, in a study of treatment naïve pediatric Crohn disease patients age was taken into account and *Pasteurellaceae* and *Neisseriaceae* were more abundant in younger patients under the age of 10.^1^ In our current study, there were no detected known pathogenic organisms of the gastrointestinal tract in higher abundance in VEO-IBD compared to pediatric IBD, potentially suggesting that the differences between these groups may be better described by the alteration in commensal organisms and the inherent difference in what is considered “commensal” in young children.

To address the question and further understand how the alteration in commensal organisms differs between IBD and VEO-IBD patients, we developed a microbial maturity index based on a model used to characterize the gut microbiota in healthy children.^23^ We aimed to determine if there was a loss of expected commensal organisms associated with more deleterious changes in the microbiota in the youngest children, in whom the immune systems and gut microbiomes are developing, as compared to older children with IBD. Using Random Forest, we generated an MMI that correlated with age in healthy children to investigate which taxa may account for some of these differences and identified 29 taxa primarily from the phyla Firmicutes and Bacteroidetes. When we applied this index to patients with VEO-IBD and pediatric IBD, we were better able to observe both the effects of age and disease on the stool microbiome (Figures 4). This was most evident among the oldest children with IBD, who were expected to have increased maturity with increased age but remained immature, as illustrated by having the lowest RMMI (Figure 4(b)). Additionally, while a subset of the VEO-IBD samples was lower than that of the age-matched healthy controls, some had higher maturity than their healthy peers, and there was more variability in the maturity index before the age of 36 months. However, because of the inherently different commensal microbial communities by age, higher maturity may point to the disease state in VEO-IBD. Altogether, the RMMI was an excellent predictor of disease status in both IBD cohorts (Figure 4(c)).

Functional analyses were then performed to further detect microbial driven disease differences between patients with VEO-IBD and pediatric IBD. We identified differences in several genes in the VEO-IBD cohort compared to pediatric IBD and HC cohorts that code for metal-binding proteins or surface-associated proteins. It is well established that some metal transporters can modulate the interaction between the gut microbiome and metabolomic effects on the host.^31^ In addition, metals such as zinc, are critical in anti-inflammatory response in the host. Consistent with this, the encoded proteins have been associated with inflammation in several disease processes, including rheumatoid arthritis and IBD. Other studies have shown that certain metals can regulate methylation or histone modification, contributing to disease progression through epigenic modification. While further investigation is ongoing and necessary to determine relevance in a larger cohort and specification of genes involved, these findings may point to some potential drivers of the disease.

This study provides a foundation for understanding the microbial community structure in children with IBD and VEO-IBD across all ages after the onset of disease. While the character of dysbiosis changes and has significant differences throughout the pediatric age range, the onset and contributing factors, immune and environmental, remain unknown. Additionally, the trajectory of dysbiosis is unknown. Collaborative longitudinal efforts are necessary and are in process, to address these questions with the goal of developing potential therapeutic strategies to modulate the microbial community structure to induce remission and mucosal healing. As an observational study, there were some inherent limitations regarding the heterogeneity of the study groups, including differences in treatment regimens and the duration of disease prior to enrollment. Due to the young age of onset and inherent immune dysregulation associated with VEO-IBD, antibiotics are often chosen as the primary therapy or in conjunction with conventional therapy. This observational study did not exclude these subjects to capture a representative sample of this patient population, but sensitivity analyses were performed to demonstrate consistent findings. Indications for antibiotic use differed between patients with VEO-IBD and age-matched healthy controls. We also did not observe any differences in the microbiome (or microbial structure) with the initiation of IBD therapy or disease activity over time. This, similar to antibiotic regimens, may be due to the variety of treatment strategies used in a limited sample size or the relatively limited longitudinal study window of 8 weeks. Controlling for treatments and expanding the follow-up period may allow for further analyses, particularly for MMI. Applying the MMI model to a larger validation cohort would strengthen our findings as would characterize the patterns of microbial communities that coexist prior to diagnosis to determine if microbial therapeutic-based interventions would be useful. Finally, recent work has implicated fungal infection of inflamed tissue as a potentially important contributor to the disease process, so it may also be useful to investigate similar cohorts using specimens of diseased tissue.^32^

Despite these limitations, this investigation demonstrates that the microbial community structure continues to change, as depicted by the MMI, independent of inflammation, beyond the first 3 years of life. In addition, we observed that the effect of inflammation in patients with VEO-IBD creates a microbial niche that differs from that in children with pediatric IBD, which may be the result of the early disruption of normal microbiome development or the different drivers of disease in VEO-IBD as compared to IBD. Further studies of stool samples from children with VEO-IBD would be useful to determine if the differences in taxa are reproducible. To determine if disease activity and therapy affect the microbiome, an inception cohort study in children with VEO-IBD is currently in progress. In the future, a large multicenter study of children before the onset of disease will undoubtedly shed light on the relevant microbial changes in IBD and whether microbial maturity can be altered in patients with IBD. With further insight, there may be a role for microbially targeted therapeutics in restoring health to the microbiome in IBD and other disease processes.

## Patients and methods

### Study participants

This prospective observational study was performed at the Children’s Hospital of Philadelphia (CHOP). The participants were aged 3 months to 19 years old. This study was approved by the Children’s Hospital of Philadelphia Institutional Review Boards (#15–011817 and 14–010826). All participants or legal guardians provided informed written consent, and when age was appropriate, assent prior to enrollment in the study. Patients included children diagnosed with IBD between the ages of 0 and 6 years (VEO-IBD) based on clinical history, esophagogastroduodenoscopy, and colonoscopy with confirmatory biopsies, laboratory studies, and radiological examinations. The VEO-IBD cohort consisted of two groups: children <7 years old at the time of sample collection and who provided longitudinal samples, and children who were diagnosed <7 years of age at the time of sample collection only, with ages ranging from 8 to 21 years. This group provided a single cross-sectional stool sample. The pediatric IBD cohort included patients diagnosed with IBD between the ages 8–21 years old who were recruited from the CHOP Pediatric IBD Center. The exclusion criteria were as follows: concurrent intestinal comorbidity including positive celiac serology and histology, Hirschsprung disease, eosinophilic esophagitis, preceding diagnosis of immunodeficiency, short bowel syndrome, and infection. Healthy control (HC) cohorts included children without IBD, gastrointestinal symptoms, or any prior chronic diagnosis who were enrolled from primary care clinics and the CHOP Emergency Department between the ages of 3 months and 20 years old. The inclusion criterion for HCs was no prior chronic diagnoses. Those patients included from the Emergency Department had been diagnosed with an acute infection such as acute otitis media, pharyngitis, or pneumonia and treated with a course of antibiotics to be enrolled as a comparison group for the children with VEO-IBD on antibiotics. The patients that were included into the HC without antibiotics were recruited from the primary care network at CHOP but had no illnesses or active symptoms at the time of enrollment. HC were excluded if they had infection, diarrhea, or a family member with documented gastrointestinal infections such as *C. difficile.*

### Sample collection

Stool samples were collected at the baseline enrollment visit and 4 and 8 weeks after the enrollment visit in the pediatric controls, pediatric IBD patients, and in 58 out of 66 VEO-IBD patients. The remaining eight VEO-IBD patients only provided one stool sample. Stool was collected only at one time point in eight patients diagnosed with VEO-IBD but were older than age 6 at the time of sampling. Patients and families were provided instructions for collecting stool at home and shipping back on ice to CHOP. Stool was aliquoted upon receipt into stool swabs, cryoextract tube, and 2 mL tube for stool calprotectin. Stool was then promptly stored in −80°C freezer.

### Clinical assessment

Demographic information and clinical disease characteristics were collected from each patient and the control group during enrollment. Disease activity was assessed using fecal calprotectin for each sample.

### Calprotectin

Calprotectin levels were measured in fecal samples using the QUANTA Lite Calprotectin Extended Range ELISA kit (Inova Diagnostics, San Diego, CA), strictly in accordance with the manufacturer’s protocol. To extract the samples, 100 mg of feces was mixed 1:50 (w/v) with extraction buffer, vortexed for 30 s, and then homogenized for 25 min on a shaker. One milliliter of the homogenate was centrifuged for 20 min at 10,000 × g, and the cleared supernatant was stored at −20°C until ELISAs were performed. ELISAs were conducted on cleared supernatants diluted 1:400 in dilution buffer. The calibrators and controls were run alongside the samples on each plate. Absorbance was measured at 450 nm using an EnSpire Multimodal Plate Reader (Perkin Elmer, Waltham, MA), and calprotectin levels were calculated based on a standard curve generated using the 4PL method.

### Metagenomic sequence acquisition

DNA was extracted from stool samples using the DNeasy PowerSoil Kit (Qiagen, Germantown, MD, USA). Sequencing libraries were generated using the NexteraXT DNA Library Preparation Kit (Illumina, San Diego, CA, USA) and sequenced on a HiSeq 2500 platform using 2 × 125 bp chemistry. To assess environmental and reagent contamination, extraction blanks and DNA-free water were processed in parallel with experimental samples. As a positive control, libraries were constructed from a laboratory-generated mock community consisting of DNA from *Vibrio campbellii* and lambda phages.

### Bioinformatic analysis of microbial communities

Shotgun metagenomic sequencing data were processed using the Sunbeam pipeline.^33^ Paired-end reads were quality-filtered and Illumina adapter sequences were removed using Trimmomatic 0.36.^34^ Low-complexity reads were marked and removed using Komplexity.^33^ Reads that aligned to the human genome (hg38) or the phiX 174 genome were removed. Taxonomic classification was performed using MetaPhlAn2.^35^ Gene ortholog abundances were estimated by alignment with the Kyoto Encyclopedia of Genes and Genomes (KEGG) database.^36^ Microbial community composition was quantified using richness and Shannon diversity as measures of alpha diversity, and abundance-weighted Bray-Curtis distance as a measure of beta diversity. We used a zero-inflated beta regression with random effects (ZIBR) model to carry out a combined test of the abundance and prevalence of taxa between study groups.^37^ Because the ZIBR test allows us to asses abundance and prevalence separately in addition to the combined test, we indicate where the tests of each component were statistically significant. For taxonomic comparisons, species present in less than 20% of the samples were removed and subjects with missing visits were excluded from ZIBR analyses. Gene ortholog comparisons were also performed using the ZIBR test and included gene. Age and antibiotic use were included as covariates in taxonomic and gene ortholog comparisons. We considered α = 0.05as the level of statistical significance. P-values were adjusted to control for a false discovery rate of 5% when multiple tests were performed.

DNA sequencing data supporting this study were deposited in the NCBI Sequence Read Archive under accession PRJNA1045596.

### Development of microbial maturity index

Random Forest (R package randomForest) was used to perform classification between pairs of groups, and variable importance scores were used to rank the variables important for classification. In each comparison, species with maximum abundance ≤ 0.01% were removed, and the Random Forest model was applied to samples at the Baseline Visit 1000 times to reduce the randomness of the Random Forest. Using Random Forests, we regressed the relative abundance of bacterial taxa against each child’s chronological age at sample collection.

We initially used 70% of the HCs without antibiotics to train random forest models to predict age (units in months) based on the microbiome data and used a nested cross-validation method to evaluate the model performance with a sequentially reduced number of species. The number of species that yielded the minimum cross-validation error was 29. We chose the top 29 species (ranked by their average importance score from the Random Forest models, which were determined over 1000 iterations). (Supplemental Table S3) We then trained the final Random Forest model and used it to predict the microbial age, which is called the Microbial Maturity Index (MMI), for the testing set and for all other samples. We also fitted a smooth spline between the MMI and chronological age of the healthy controls using the testing set. The relative MMI was calculated for each sample as follows: RMMI = MMI of the sample – predicted MMI from the spline fit of healthy controls of the same age.^23^ We used a mixture modeling approach implemented in FlexMix to build a two-component model, where one component was constrained to be a healthy fit.^38^ This was used to divide the samples into two groups: healthy-like vs. non-healthy. Clinical variables associated with and measures of diversity from individual samples were compared between groups.

## Supplementary Material

Supplemental Table 2A_VEOvIBDKegg.csv

Supplemental Figure 3.jpg

Supplemental Table 2B HCltvsVEOKegg.csv

Supplemental Table 1B_HCgreater7vsIBD.csv

Supplemental Figure 5.jpg

Supplemental Table 1C_VEOvsIBD.csv

Supplemental Table 2C HCgreater7vsIBDKegg.csv

Supplemental Figure 1.jpg

Supplemental Figure 2.jpg

Supplemental Table 3_MMItaxa.csv

Supplemental Figure 4.jpg

Supplemental Figure 6.jpg

Supplemental Table 1A_HClt7vsVEO.csv

## Abbreviations

BC: Bray Curtis
HC: healthy controls
IBD: inflammatory bowel disease
MMI: microbiota maturity index
PTI: children diagnosed with VEO-IBD but provided samples after age 7
RMMI: Relative microbiota maturity index
SD: Shannon Diversity index
VEO-IBD: very early onset inflammatory bowel disease
