# Supplementary figures and images for: The intestinal microbiome of inflammatory bowel disease across the pediatric age range
Source: Gut Microbes. 2024 Feb 25;16(1):2317932. doi: 10.1080/19490976.2024.2317932 (PMC10900269; doi:10.1080/19490976.2024.2317932)

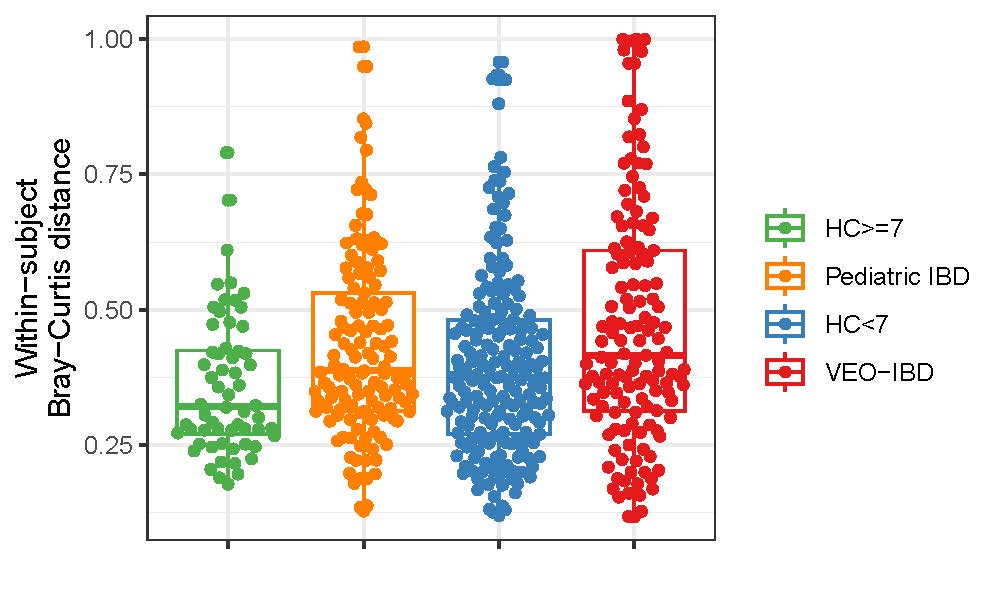

Supplement: Supplemental Figure 3.jpg [file KGMI_A_2317932_SM3096.jpg]

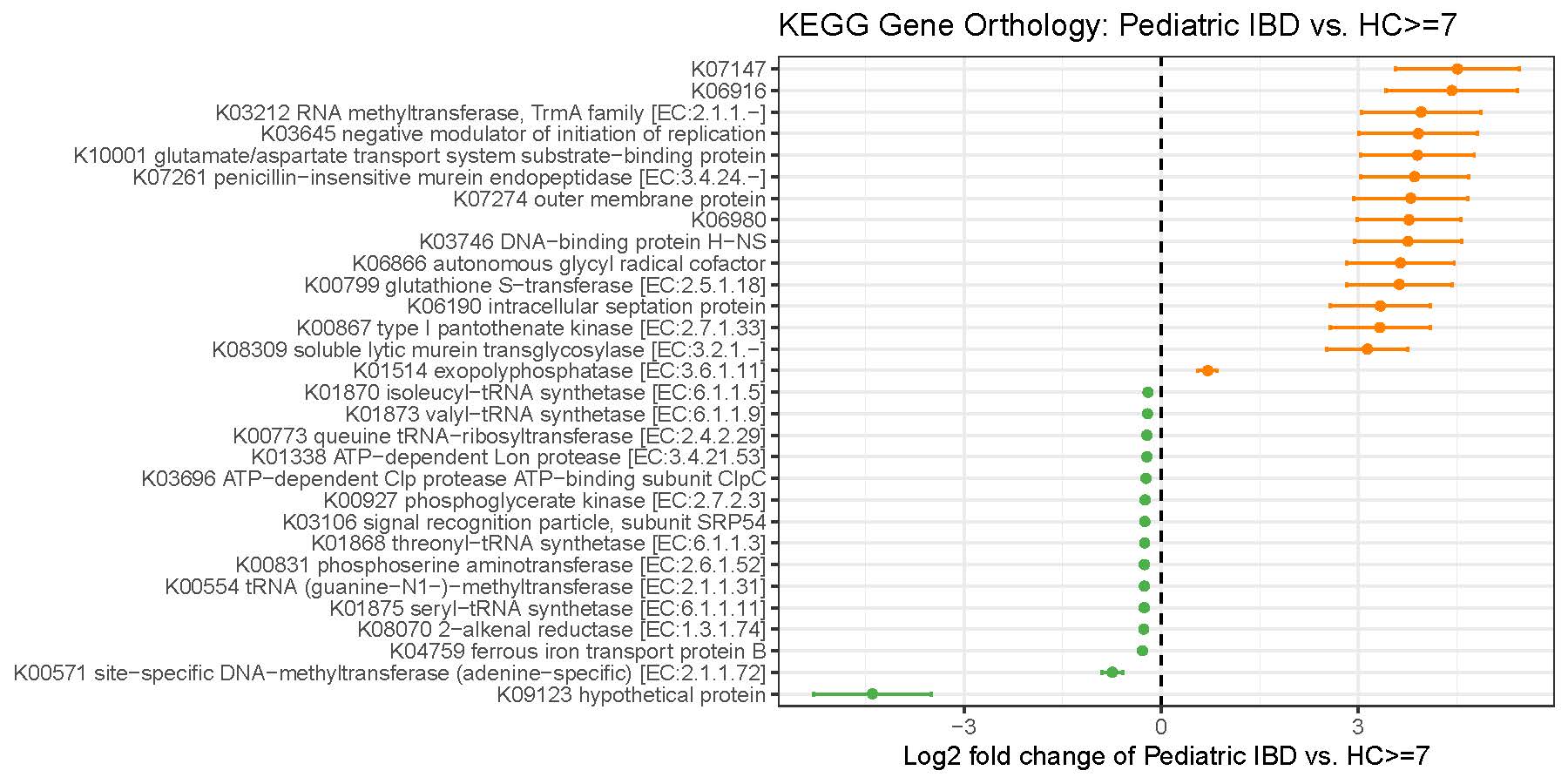

Supplement: Supplemental Figure 5.jpg [file KGMI_A_2317932_SM3093.jpg]

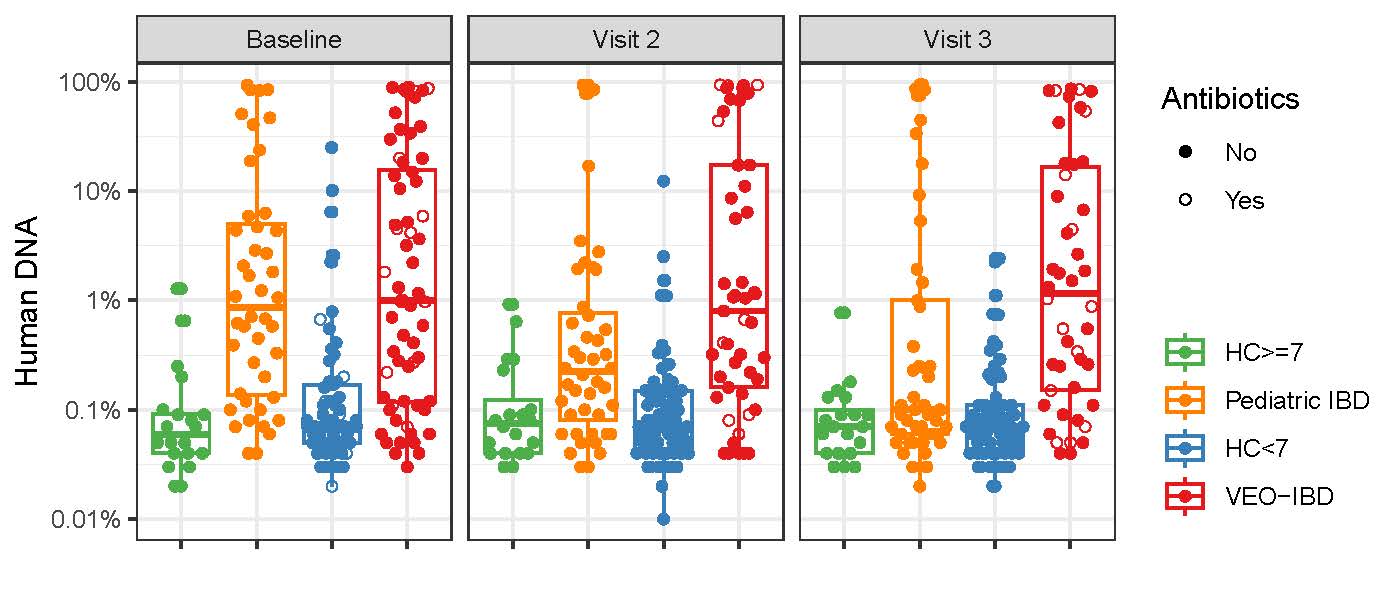

Supplement: Supplemental Figure 1.jpg [file KGMI_A_2317932_SM3090.jpg]

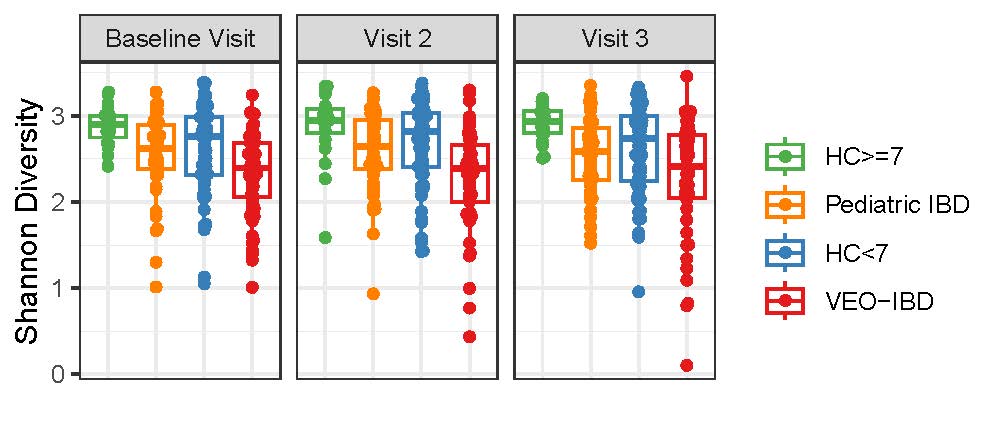

Supplement: Supplemental Figure 2.jpg [file KGMI_A_2317932_SM3089.jpg]

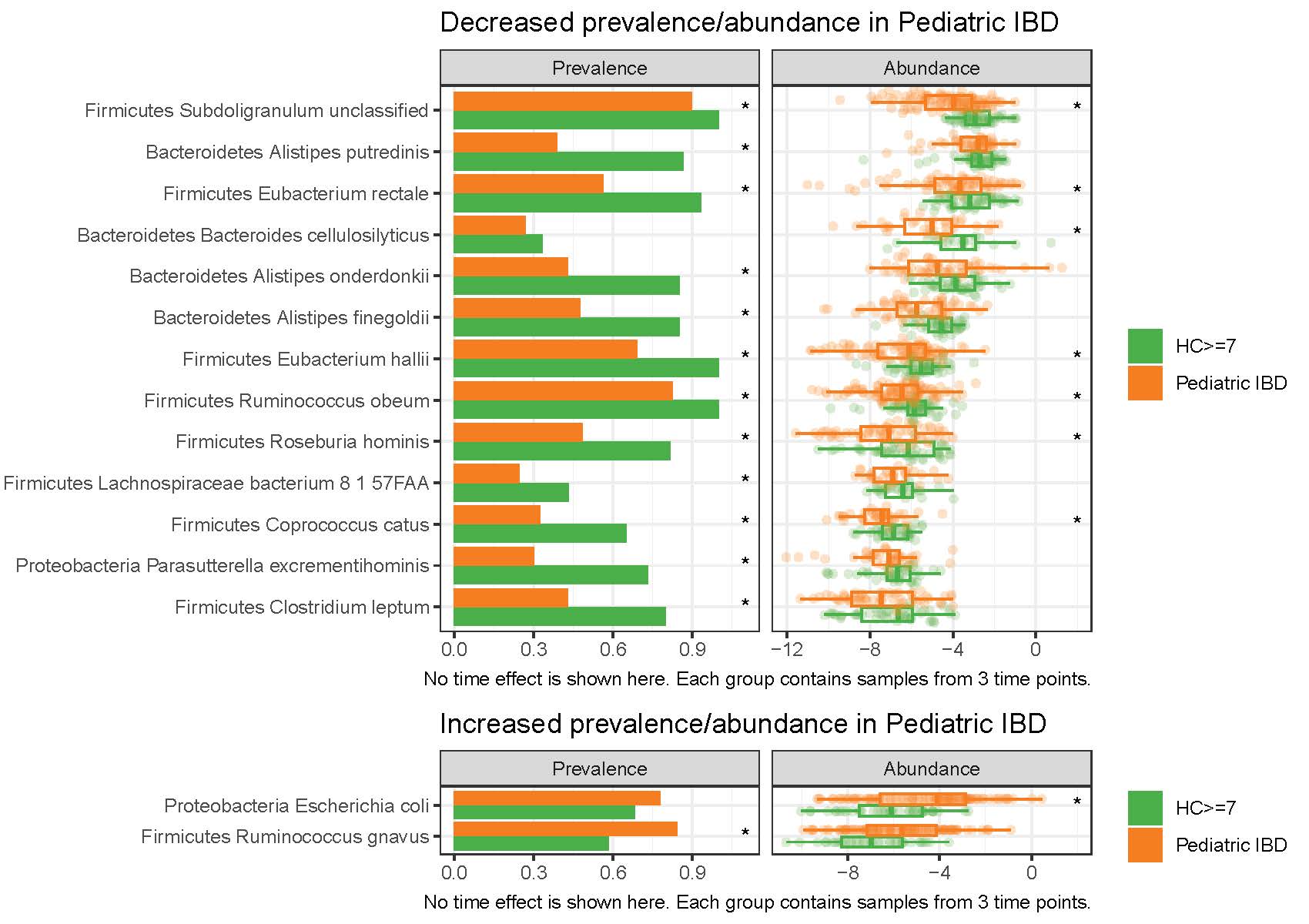

Supplement: Supplemental Figure 4.jpg [file KGMI_A_2317932_SM3087.jpg]

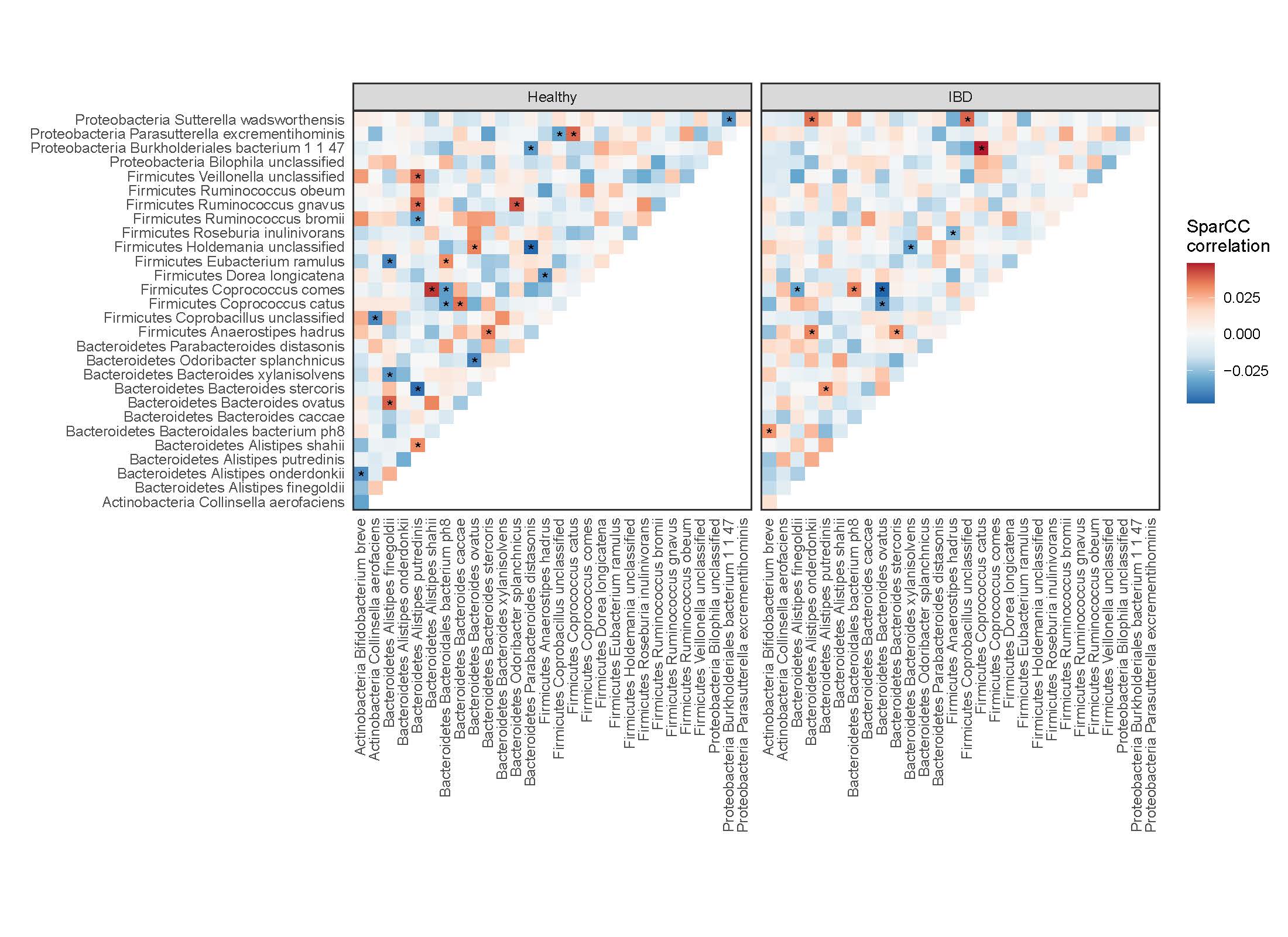

Supplement: Supplemental Figure 6.jpg [file KGMI_A_2317932_SM3086.jpg]
